# The spleen is the graveyard of CD4+ cells in patients with immunological failure of visceral leishmaniasis and AIDS

**DOI:** 10.1186/s13071-024-06151-6

**Published:** 2024-03-15

**Authors:** Luis Gustavo Cavalcante Reinaldo, Raimundo José Cunha Araújo Júnior, Thiago Melo Diniz, Rafael de Deus Moura, Antônio José Meneses Filho, Caio Victor Verçosa de Macedo Furtado, Washington Luis Conrado dos Santos, Dorcas Lamounier Costa, Kelsen Dantas Eulálio, Gabriel R. Ferreira, Carlos Henrique Nery Costa

**Affiliations:** 1 University Hospital of the Federal University of Piauí, Teresina, Brazil; 2 Hospital Getúlio Vargas, Teresina, Brazil; 3 Department of Community Medicine, Federal University of Piauí, Teresina, Brazil; 4 Department of Community Medicine, Federal University of Piauí, Teresina, Brazil; 5 Oswaldo Cruz Foundation, Gonçalo Moniz Institute, Salvador, Brazil; 6 Maternal and Child Department, Federal University of Piauí, Teresina, Brazil; 7 Instituto de Doenças Tropicais Natan Portella, Teresina, Brazil; 8 Department of Microbiology-Infectious Disease and Immunology, Faculty of Medicine, University Laval, Laval, QC Canada; 9 Intelligence Center for Emerging and Neglected Tropical Diseases, Teresina, Brazil

**Keywords:** Visceral leishmaniasis, Kala-azar, *Leishmania infantum*, Hypersplenism, AIDS, T-lymphocytes, CD4+, CD8+, Macrophages, Immunological failure

## Abstract

**Background:**

Visceral leishmaniasis (VL), or kala-azar, is a common comorbidity in patients with AIDS in endemic areas. Many patients continue to experiences relapses of VL despite virological control, but with immunological failure. These patients remain chronically symptomatic with hypersplenism, for example with anemia, leukopenia, and thrombocytopenia, and are at risk of severe co-infection due to low CD4+ count. Therefore, in this study, splenectomized patients with VL and HIV infection were investigated to understand why the CD4+ count fails to recover in these patients, evaluating the importance of spleen mass for hypersplenism and immunological failure.

**Methods:**

From a retrospective open cohort of 13 patients who had previously undergone splenectomy as salvage therapy for relapsing VL, 11 patients with HIV infection were investigated. This study compared the patients’ complete blood cell count (CBC) and CD4+ and CD8+ cell counts before and after splenectomy with respect to spleen weight.

**Results:**

CBC was substantially improved after splenectomy, indicating hypersplenism. However, to the best of our knowledge, this is the first study to show that spleen mass is strongly and negatively correlated with CD4+ cell count (*ρ* = −0.71, *P* = 0.015).

**Conclusions:**

This finding was unexpected, as the spleen is the most extensive lymphoid tissue and T-lymphocyte source. After reviewing the literature and reasoning, we hypothesized that the immunological failure was secondary to CD4+ loss initially by apoptosis in the spleen induced by productive HIV infection and, subsequently, by pyroptosis sustained by parasitic infection in spleen macrophages.

**Graphical Abstract:**

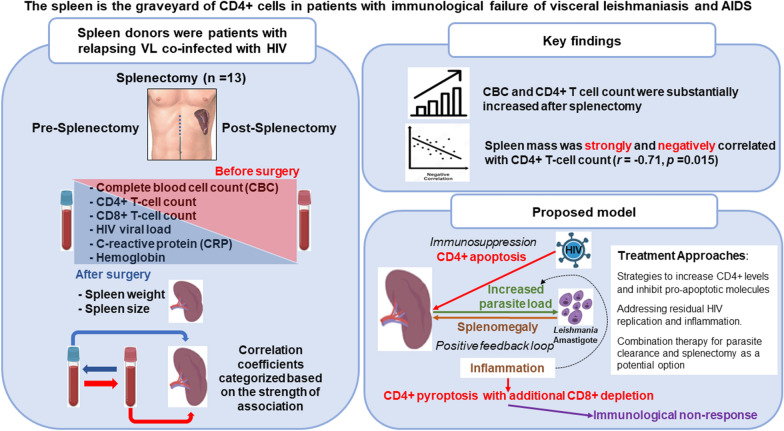

**Supplementary Information:**

The online version contains supplementary material available at 10.1186/s13071-024-06151-6.

## Background

Visceral leishmaniasis (VL), or kala-azar, is responsible for significant morbidity in tropical and subtropical areas. The viscerotropic protozoa *Leishmania infantum* and *Leishmania donovani* are intracellular parasites of the mononuclear lineage called amastigotes. They are transmitted by sand flies and distributed mainly in the vertebrate host's spleen, liver, and bone marrow [1].

VL is also a common comorbidity in patients with HIV in endemic regions. Patients with low CD4+ cell counts in the first episode of VL develop frequent relapses that, despite the use of appropriate medications [2], persist chronically, with VL symptoms such as anemia, cachexia, hepatosplenomegaly, and decreased blood cell counts [3]. Importantly, even patients with virological control of HIV experience immunological failure, with very low CD4+ cell counts, and are at increased risk of opportunistic infections, successive hospitalizations, and death.

Splenectomy rescue therapy was previously performed in 13 patients with VL, with and without HIV infection, who were not responsive to secondary prophylaxis to VL as a salvage therapy, as splenectomy may be curative for patients with relapsing VL without immunosuppression [4]. This communication adds information but is now restricted to the 11 patients with HIV infection who underwent the surgical procedure. The study aims to describe the relationships between pre- and post-splenectomy complete blood count (CBC), and in particular CD4+ and CD8+ cell counts, with respect to the spleen mass as a way to understand the role of an enlarged spleen in relapses and immunological failure in co-infected patients.

## Methods

Spleen donors were part of an open cohort study involving individuals with VL unresponsive to drug treatment, co-infected or not with HIV, followed in the Institute of Tropical Diseases “Natan Portella” in Teresina, Piauí, Brazil, from 2008 to 2019. The eligibility criteria for surgery were described elsewhere [4]. The last routine laboratory tests before splenectomy were registered as followed: CBC, blood CD4+ and CD8+ cell counts, HIV viral load, and C-reactive protein (CRP). Hemoglobin was used as a proxy for red cell count. Cell counts after surgery were also registered. The splenectomies were performed at the University Hospital of the Federal University of Piauí (HU-UFPI) and Hospital Getúlio Vargas (HGV) in Teresina, Brazil. Spleens were measured and weighed immediately after the surgery.

Pearson’s and Spearman’s correlation tests for spleen weight with pre- and post-splenectomy CD4+ and CD8+ cell counts, CBC, and CRP were analyzed when indicated using Stata 15.1 software (StataCorp LLC, College Station, TX, USA). Because of the small sample size, statistical significance in this study was achieved when there was a strong correlation coefficient, e.g., a value of approximately *ρ* > 0.6. Therefore, following standardized verbal and informal definitions, coefficients were assumed to have a robust correlation if they were > 0.6, moderate if > 0.4 to 0.6, weak if > 0.2 to 0.4, and no association if they were 0.2 or less [5].

## Results

Table 1 shows the primary patient data. There were 10 men and one woman; ages varied from 33 to 58 years, with a mean of 41.9 years. Spleen measurements were as follows: length of 12–27 cm, width of 8–17 cm, and thickness of 4–9 cm. Spleen weight varied from 295 to 1882 g, with a mean of 784 g (95% confidence interval (CI): 295–1882) and a median of 650 g (interquartile range: 550–1042). Patients were anemic and had leukopenia and thrombocytopenia, with mean hemoglobin of 8.0 g/dl, mean leucocytes of 2190 cells/ml, and mean platelets of 126,474/ml. CRP was low in seven patients and slightly elevated in two, with a mean of 4.1 mg/l. CD4+ T-cell count was measured from 9 to 323 days before splenectomy and ranged from 33 to 104 cells/μl, with a mean of 72 cells/μl and median of 73 cells/μl. CD8+ T-cells varied from 177 to 638 cells/μl, with a mean of 375 cells /μl and a median of 344 cells/ml. Viral load was undetectable in all patients. Additional file 1: Table S1 shows the dates and time elapsed between the last CD4+ count pre-splenectomy and the first count post-splenectomy. Additionally, the number of years between the diagnosis of HIV/AIDS and of VL up to splenectomy is shown in Additional file 2: Table S2, providing insight into the number of relapses experienced by each patient before splenectomy was performed.

**Table 1:** Characteristics and blood tests of 11 patients with relapsing visceral leishmaniasis and AIDS who underwent splenectomy

Characteristic	Mean	95% CI	Median	Interquartile range
Age (years)	41.9	36.0–47.8	36.0	35.0–51.0
Male (%)	90.9	0.59–100.0	–	–
Female (%)	9.1	0.00–0.41	–	–
Spleen weight (g)	783.9	496.8–1071.0	650.0	550–1042
CD4+ T-cells (cells/µl)	72.0	55.8–88.2	73	50–93
CD8+ T-cells (cells/µl)	374.9	275.3–474.5	344	295–557
Hemoglobin (g/dl)	8.0	7.1–8.9	8.3	6.6–9.0
Leucocytes (cells/µl)	2190	1644.6–2735.4	2200	1430–2730
Platelets (platelets/µl)	126,472.7	98,965.8–153,979.7	120,000	93,000–150,000
C-reactive protein (mg/l)	4.1	1.3–6.8	3.2	2.4–3.4

**Fig. 1:**
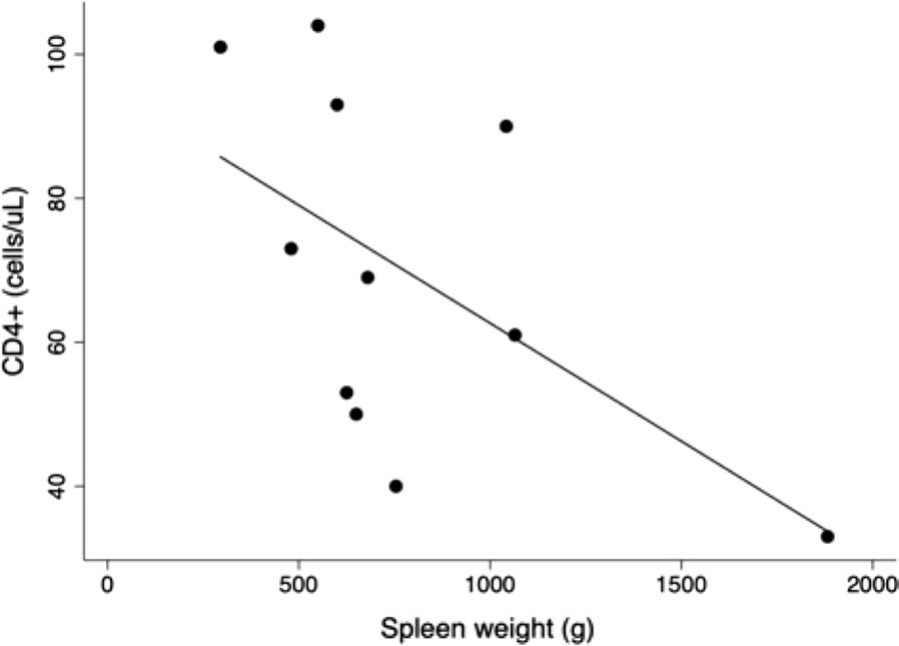
Correlation of spleen weight and CD4+ cell count of 11 splenectomized patients with immunological failure due to visceral leishmaniasis and AIDS (*ρ* = −0.71, *P* = 0.015)

Because the spleen weight was not normally distributed, the Spearman correlation test was performed to compare CBC, CD4+, and CD8+ with spleen weight. Table 2 shows the correlation analysis of spleen weight, and Fig. 1 illustrates the correlation of spleen mass with blood CD4+ cells. There was a weak negative correlation of CBC with hemoglobin (*ρ* = −0.38), a moderate negative correlation with leucocytes (*ρ* = −0.52, *P* = 0.103), and no correlation with platelet count (*ρ* = −0.16). There was no correlation between spleen weight and CRP levels. There was a strong negative, statistically significant correlation between the pre-splenectomy CD4+ count and spleen weight (*ρ* = −0.71, *P* = 0.015) but no correlation of spleen mass with blood CD8+ (*ρ* = 0.07).

**Table 2:**
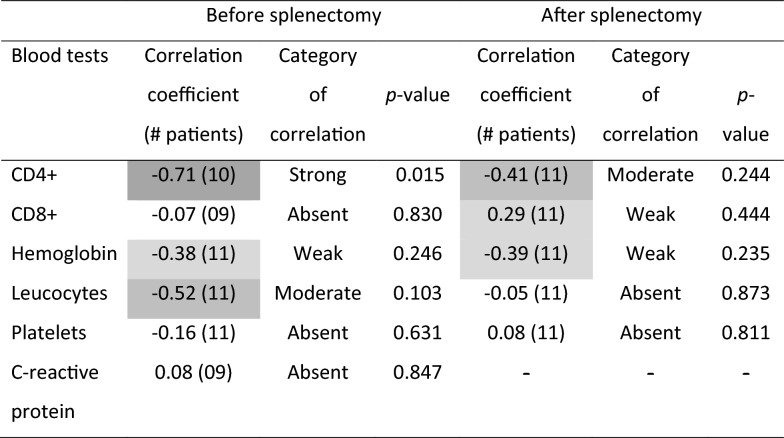
Spearman’s correlation coefficients between spleen weight and CD4+ and CD8+ counts, hemoglobin, leukocytes, platelets, before and after splenectomy, and C-reactive protein levels in patients who underwent splenectomy with relapsing visceral leishmaniasis and AIDS

Table 3 shows the Pearson’s correlation coefficient of CD4+ and CD8+ lymphocytes, hemoglobin, leucocytes, and platelets. All counts increased after splenectomy at variable grades. Although hemoglobin increased 1.3 times, patients were still anemic, and the correlation between pre- and post-surgical measurements was weak (*ρ* = 0.30). Leucocytes increased 5.4 times from leukopenia to leukocytosis levels, and pre- and post-surgical values were strongly correlated (*ρ* = 0.72, *P*-value = 0.013). Platelets increased 2.3 times, and the two values had a moderate correlation (*ρ* = 0.44). After splenectomy, CD4+ increased 3.9-fold, reaching a mean of 278.1 cells/μl, with values ranging from 135 to 614 cells/μl. The correlation coefficient with the antecedent measurement was 0.43. CD8+ cells increased 2.9-fold and were weakly correlated with pre-splenectomy values.

**Table 3:**
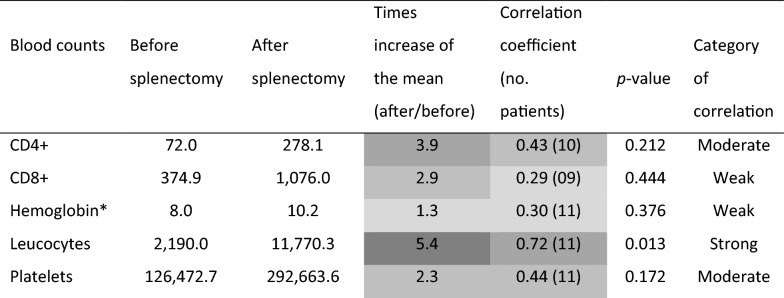
Pearson’s correlation coefficients before and after splenectomy of hemoglobin, leukocytes, platelets, and CD4+ and CD8+ counts of 11 patients with relapsing visceral leishmaniasis and AIDS who underwent splenectomy

*One patient received red cell transfusion immediately after splenectomy

## Discussion

This study describes the unexpected strong, negative association of spleen weight with CD4+ cell count in splenectomized patients with VL and AIDS and less expressive changes with CBC. To the best of our knowledge, this association between spleen mass and CD4+ count is the first to be described in VL and HIV infection, in either humans or experimental animals, although a previous study in Uganda had observed a slightly lower CD4+ cell count in healthy, HIV-negative individuals with larger spleens, as determined by palpation [6]. The present observation was made in the context of patients with VL and AIDS with very large spleens, with virological control but with immunological failure, and who underwent splenectomy, as previously reported. One patient had longer elapsed time between the low last CD4+ count and splenectomy. However, as he was in a new relapse, it can be inferred that the low count was stable over time, and therefore it is unlikely that the conclusion would have differed if the CD4+ count had been performed closer to the surgery date.

The human spleen architecture and function are divided into red and white pulps, and between them, the perifollicular zone. The observed low CBC counts were secondary to hypersplenism. In this condition, the red pulp of enlarged spleens sequesters, forming elements of the blood [7]. The correlation of reduced CBC with spleen mass was negative but only weak or moderate, possibly because of other pathogenetic mechanisms of VL [8]. Anemia had a more modest and slower recovery as it had a much longer recovery time than neutrophils and platelets. In contrast, the last two cellular elements had a fast recovery. They increased above normal levels, as noted previously [9, 10].

The most critical question of this study is why *L. infantum* was not controlled in our patients with AIDS, despite the anti-*Leishmania* treatment and combined antiretroviral therapy (cART). However, some immunocompetent patients fail when parasite resistance is not detected [11, 12]. Two patients of this cohort of initially 13 individuals who were not HIV-infected endured this enigmatic situation and were cured after splenectomy [4].

All patients had low CD4+ count, a pervasive observation in relapsing VL with AIDS [13]. CD4+ T-cells are the central mediators of immune response [14]. Low CD4+ is usually due to a lack of HIV treatment or treatment failure [15]. The mechanisms involved in the decreased CD4+ in HIV-infected patients without cART include the cytopathic effects of HIV in 5% of productively infected cells and 95% of non-productively infected bystander cells before cART is initiated. This process is followed by a hyperactive state of the immune system characterized by high non-specific T-cell activation, proliferation, and turnover [16–19]. Even in patients with HIV without co-infections, CD4+ remains low despite being under virological control, for example, immunological failure or immunological non-response [20]. More recent studies propose that reduced thymic function and increased immune activation, followed by T-cell exhaustion and senescence, are the primary drivers of poor CD4+ T-lymphocyte reconstitution and immunological failure [21, 22]. However, several factors have been proposed to lead to immunological non-response, including nadir CD4+ count and the age at initiation of cART, among others [23, 24].

Relapses of VL are much more frequent in HIV-infected patients, which was evidenced by the fact that 11 of 13 co-infected patients experienced relapsing VL in this cohort, although less than 10% of VL patients have HIV infection in Brazil [3]. There are several reasons for VL relapses in patients with HIV. The most important seems to be the low CD4+ at VL diagnosis, as it predicts VL relapses [25], suggesting that an early poor protective host response before treatment allows parasite proliferation, which becomes uncontainable. However, our patients were experiencing low CD4+ counts and relapses while productive HIV infection was under control. The reason for this phenomenon, therefore, is critical component, as amastigote survival and relapses are made easier by the low CD4+ levels, which are unable to drive effective Th1 immune response against the parasite. Observations indicate that this immunological failure in patients with VL is driven by chronic immune activation followed by low thymic output and CD4+ and CD8+ exhaustion [26, 27].

The spleen is a productive source of CD4+ cells [28, 29]. The white pulp is composed of diverse cellular populations organized in the periarteriolar lymphoid sheaths (PALS), lymphoid follicles, and marginal zone, including CD4+, CD8+, and natural killer (NK) lymphocytes, monocytes, macrophages, dendritic cells, and plasma cells. The white pulp accounts for one fourth of the spleen mass and one fourth of the body’s lymphoid system [7, 30–34]. Therefore, hypothetically, the larger the spleen, the greater the number of circulating CD4+ cells, contrary to what was observed.

The spleen is highly parasitized during VL without HIV infection [35–37], including during relapses [36, 38], and splenectomy is curative in patients without immunosuppression and improves those with HIV co-infection [4, 39, 40]. The importance of the spleen for the observed CD4+ drop is highlighted by the prolonged effect of splenectomy, as the CD4+ count remained correlated with previous spleen size after splenectomy. Moreover, severe lymphopenia is a sign of severe VL and is observed in patients with VL independently of HIV infection [8]. Therefore, the spleen is critical for CD4+ drop during VL and seems crucial for relapses. The spleen is also critical in HIV infection. Patients with AIDS who underwent splenectomy had an increase in CD4+ number, and paradoxically, the CD4+ population was higher in the spleen of patients with HIV [41–43]. Finally, it has been shown that the spleen is the sanctuary for HIV during cART by clonal expansion and maintaining a large pool of proviruses [33]. Therefore, viscerotropic *Leishmania* and HIV meet in the spleen, and their coexistence explains why the spleen mass is so strongly correlated with peripheral CD4+ count. The large spleens of VL and AIDS patients highlight the importance of this co-localization with respect to the sharp decrease in CD4+. Because the average weight of the spleen in the patients with VL and AIDS observed in this study was over five times that in the non-diseased population (784 g versus 139 g) [44], the mean contribution of the spleen lymphoid tissue to the total number of lymphocytes has to be much higher than the one-fourth contribution in non-diseased patients [30], reaching 65% in average spleens and up to more than 80% in the heaviest spleen of this series. Therefore, the spleen lymphoid tissue acquired a critical role in lymphocyte count. Hence, it may be concluded that what happened in the spleen critically influenced the immune system of the patients with VL and AIDS.

Increased lymphocyte count is observed in infections involving splenomegaly and the lymphoid system, including acute HIV infection [45, 46]. However, instead of increasing T-lymphocyte proliferation, VL and AIDS comorbidity cleared the spleen of CD4+ cells that otherwise would be released for circulation. The conclusion from the data is obvious: the spleen is the “graveyard” of CD4+ cells in patients with relapsing VL and AIDS. Indeed, depopulation from PALS, where spleen T-lymphocytes are concentrated, is observed in patients with AIDS without VL [47]. The effect of splenectomy on increasing CD4+ is thus to free CD4+ cells from mass destruction in the spleen.

The most likely explanation for the immunological failure of patients with VL and AIDS seems to be the remarkable pro-inflammatory status of VL, with a high concentration of the circulating cytokines linked to the disease’s pathogenicity [8, 48]. This process is sustained by pervasive macrophage and dendritic cell parasitic infection throughout all spleen regions, from the red pulp, marginal zone, and lymphoid follicles to the PALS, where T-lymphocytes are concentrated [37]. The macrophage-led spleen inflammatory status, as seen in one non-HIV-infected patient [38], may release signs of transactivation of provirus-infected CD4+ driven by the innate immune response to viscerotropic *Leishmania*, allowing viral replication and inducing infected and bystander T-cell apoptosis and pyroptosis [17, 49]. Possible complementary mechanisms are the white pulp disorganization promoted by advanced VL [35, 37, 50] and transmission of provirus and incomplete HIV DNA from infected CD4+ and macrophages to resting CD4+ cells [17, 19, 49, 51–54].

Contrary to CD4+, CD8+ cell count was not correlated with spleen mass in our patients. This finding is likely due to the independence of CD8+ from the spleen, as observed in mice compared to CD4+ [55]. On the other hand, before splenectomy, CD8+ counts were at their lower limits of normality, indicating that the large and inflamed spleen also led to some CD8+ loss, as observed in the peripheral blood, in line with the lower CD8+ counts in patients with VL before treatment [50]. Accordingly, CD8+ cells may become activated and exhausted due to the proximity of the inflamation to the amastigote-infected macrophages [26] with CD4+ in the PALS [34, 37]. Indeed, reduced CD8+ count has already been observed at the advanced stages of viscerotropic *Leishmania* and HIV co-infection [26, 56]. Hence, it seems reasonable that decreased CD8+ count may represent the last drop necessary for the final stage of the immunological failure to control parasite proliferation in relapsing VL.

Developing approaches to overcome the immunological failure that perpetuates VL relapses in HIV-infected patients is essential to restoring health and improving survival. An immunological approach could include drugs to increase CD4+ levels (interleukin [IL]-2, IL-7) or inhibit pro-apoptotic molecules, such as PD-1/PD-1L and CTLA-4/CD28. Residual HIV replication is seen as an additional mechanism driving immunological failure. Because most patients were treated before the introduction of the more potent antivirals CCR5 and integrase inhibitors, these drugs are proper candidates for testing the hypothesis to improve the prognosis of co-infected patients. Anti-inflammatory drugs such as steroids, mesalamine, and statins are also under evaluation. To enhance parasite clearance, combination therapy of antiparasitic treatments is now recommended for East Africa and India and may also be used in areas of *L. infantum* transmission, which holds promise for these patients [57, 58]. Finally, splenectomy has been tried and seems beneficial, but it is also risky [4]. Hence, there are some hopeful alternatives for a condition that dramatically worsens the quality of life of persons living with HIV in areas where VL is endemic.

## Conclusions

Despite the small sample and the lack of sophisticated analysis and pathology data for this study, the observations described herein shed light on immunological failure and relapsing VL. Initially unnoticed HIV infection likely drives low CD4+ count, allowing the development of VL. After that, amastigote proliferation inside macrophages increases the spleen size and splenic inflammation. Meanwhile, the host's inflammatory immune response to the parasite depletes CD4+ cells, establishing a positive feedback loop that allows parasite replication despite HIV control. In later stages, CD8+ depletion could weaken the control of infected macrophages, driving the disease to severe immunological failure at its final stage. These hypotheses, however, need to be tested with appropriate molecular and histopathological studies. Our stored spleen and serum samples will help to clarify these crucial issues for patients with a poor prognosis and without a consolidated treatment strategy, providing hope for new treatments and approaches.

### Supplementary Information

**Additional file 1: Table S1.** Dates of CD4+ count and of splenectomy.

**Additional file 2: Table S2.** Elapsed time between the diagnosis of visceral leishmaniasis and HIV/AIDS and splenectomy.

## Data Availability

The full dataset was published as additional file material of reference #4, but as a small change was introduced, the most recent version can be requested from the corresponding author.
