# Supplementary material for: The spleen is the graveyard of CD4+ cells in patients with immunological failure of visceral leishmaniasis and AIDS
Source: Parasit Vectors. 2024 Mar 15;17:132. doi: 10.1186/s13071-024-06151-6 (PMC10941596; doi:10.1186/s13071-024-06151-6)
Supplement: Supplementary file 1 — Additional file 1: Table S1. Dates of CD4+ count and of splenectomy. [file 13071_2024_6151_MOESM1_ESM.docx]

Additional file 1: Table S1. Dates of CD4+ count and dates of splenectomy.

| Patient ID | Date of the **last** CD4+ count (cells/μL) **before** splenectomy | Date of splenectomy | Days between pre-splenectomy CD4+ count and splenectomy | Date of the **first** CD4+ count (cells/μL) **after** splenectomy |
| --- | --- | --- | --- | --- |
| 2 | 2013/12/12 | 2014/05/06 | 145 | 2015/06/09 |
| 3 | 2014/04/07 | 2014/06/17 | 71 | 2015/02/20 |
| 4 | 2014/12/07 | 2015/01/20 | 35 | 2015/02/13 |
| 6 | 2015/09/09) | 2016/09/15 | 9 | 2016/04/27 |
| 7 | 2016/02/19 | 2016/03/16 | 20 | 2016/06/15 |
| 8 | 2015/11/12 | 2016/09/30 | 323 | 2017/01/10 |
| 9 | 2016/05/14 | 2016/11/22 | 192 | 2016/12/09 |
| 10 | 2016/10/16 | 2016/12/08 | 63 | 2017/08/22 |
| 11 | 2017/10/02 | 2017/10/31 | 29 | 2017/12/04 |
| 12 | 2017/12/13 | 2018/01/25 | 43 | Not done* |
| 13 | 2018/04/10 | 2018/05/08 | 28 | 2018/09/07 |

*Early post-surgical death.
