# Supplementary material for: The spleen is the graveyard of CD4+ cells in patients with immunological failure of visceral leishmaniasis and AIDS
Source: Parasit Vectors. 2024 Mar 15;17:132. doi: 10.1186/s13071-024-06151-6 (PMC10941596; doi:10.1186/s13071-024-06151-6)
Supplement: Supplementary file 2 — Additional file 2: Table S2. Elapsed time between the diagnosis of visceral leishmaniasis and HIV/AIDS and splenectomy. [file 13071_2024_6151_MOESM2_ESM.docx]

Additional file 2: Table S2. Elapsed time between the diagnosis of visceral leishmaniasis and HIV/AIDS and splenectomy.

| Time elapsed between the diagnosis of HIV/AIDS and splenectomy (years) | Time elapsed between the diagnosis of VL and splenectomy (years) |
| --- | --- |
| 10 | 6 |
| 8 | 7 |
| 7 | 3 |
| 9 | 5 |
| 2 | 2 |
| 10 | 2 |
| 9 | 1 |
| 4 | 4 |
| 15 | 4 |
| 7 | 3 |
| 16 | 3 |
